# Early postoperative complications have long-term impact on quality of life after restorative proctocolectomy

**DOI:** 10.1097/MD.0000000000003966

**Published:** 2016-07-08

**Authors:** Andrew McCombie, Yun Lee, Rutvik Vanamala, Richard Gearry, Frank Frizelle, Emma McKay, Jonathan Williman, Tim Eglinton

**Affiliations:** a University of Otago, Christchurch, New Zealand; b Canterbury District Health Board, Christchurch, New Zealand.

**Keywords:** complications, ileal pouch-anal anastomosis, quality of life, restorative proctocolectomy

## Abstract

**Introduction:**

Early postoperative complications graded according to the Clavien–Dindo classification system have not previously been correlated with long-term quality of life outcomes in patients who have had restorative proctocolectomy with ileal pouch-anal anastomosis. This study aimed to assess the severity of early postoperative complications and compared these in terms of the long-term quality of life after restorative proctocolectomy in a population-based cohort of patients (operated on from 1984 to 2013). It was hypothesized that those who experienced grade 3 or 4 Clavien–Dindo complications would have worse quality of life at follow-up.

**Methods:**

This population-based study used a combination of a retrospective note review and a cross-sectional questionnaire. All patients with a restorative proctocolectomy performed in 1984–2013 in the Canterbury region were recruited using multiple sources. Early (≤30 days) and late (>30 days) complication rates were obtained via patient records. Early postoperative complications were graded according to the Clavien–Dindo classification. Quality of life was measured using the inflammatory bowel disease questionnaire.

**Results:**

One hundred and thirty-six people were identified with a median follow-up of 12 years. Data were available for 121 patients for early complications and 112 for late complications. Eighty-one eligible participants had their quality of life assessed (86% response rate). Early complications occurred in 26% and 76% had late complications. Those who had Clavien–Dindo grade 3 or 4 early complications had lower quality of life scores (*P* = 0.001) as did females (*P* = 0.004) and those with a stricture (*P* = 0.031).

**Conclusion:**

This population-based study with long-term follow-up demonstrates that Clavien–Dindo grade 3 and 4 postoperative complications are important in determining quality of life in the long term. The reduction in these complications should be a focus of patient management, as it should improve long-term quality of life.

## 1 Introduction

Restorative proctocolectomy with ileal pouch-anal anastomosis (IPAA) is commonly indicated in patients unresponsive to medical treatment with ulcerative colitis. It has also found a place in selected patients with indeterminate colitis and familial adenomatous polyposis (FAP),^[1–6]^ and is controversially used by some in patients with colonic Crohn's disease.^[6–8]^

While restorative proctocolectomy with IPAA has the advantage of maintaining anal continence and avoiding a permanent stoma, there are associated short- and long-term morbidities. A large follow-up study of 3707 restorative proctocolectomy with IPAA recipients (with a median follow up of 84 months) in Cleveland reported 34% early postoperative complications, 29% late complications (excluding pouchitis), and 5% pouch failure.^[6]^ Similarly, a study performed in Minnesota^[9]^ reported 6% failure at 10 years and a UK study^[4]^ reported 16% failure at 10 years. However, these are high-volume quaternary referral centers rather than population-based cohorts.

The morbidity associated with restorative proctocolectomy with IPAA has the potential to impact on patient quality of life (QoL). QoL can be defined as a person's self-evaluation of their present level of functioning in day-to-day living and satisfaction with it as compared to what they perceive to be optimal.^[10]^ The inflammatory bowel disease questionnaire (IBDQ)^[11]^ is commonly used for measuring QoL in ulcerative colitis and Crohn's disease patients and has been used in restorative proctocolectomy with IPAA recipients.^[12–14]^

Early postoperative complications may have long-lasting impacts on QoL. It has previously been suggested that pelvic sepsis is associated with pouch failure^[15]^ and poorer QoL.^[15,16]^ The Clavien–Dindo classification, which contains 5 grades (ranging from 1—minor deviation from normal postoperative course to 5—death), is useful for classifying early postoperative complications based on severity.^[17,18]^ No study has attempted to link the grading of complications based on the Clavien–Dindo classification to subsequent QoL in restorative proctocolectomy with IPAA recipients.

This study aims to determine the impact of early postoperative complications on long-term QoL, in particular to determine whether severe early postoperative complications (Clavien–Dindo grades 3 and 4) predict impaired QoL at long-term follow-up.

## 2 Method

### 2.1 Participants

This population-based cohort study aimed to recruit all patients with a restorative proctocolectomy with IPAA in the Canterbury region. Canterbury has an area of 45,346 km^2^.^[19]^ A June 2012 estimate of the population of Canterbury region is 558,800.^[20]^

All patients with restorative proctocolectomy with IPAA performed during the study period of 1984 to June 2013 were eligible for inclusion. Those less than 16 years of age were excluded from the study. Patients were excluded from early complications if perioperative or early postoperative notes specific to the restorative proctocolectomy with IPAA were unobtainable and from the late complications if long-term follow-up data were unobtainable. Patients were not included in the IBDQ component of the study if they were deceased or uncontactable. Participants were recruited and procedures were followed in accord with the ethical standards of the Helsinki Declaration of 1975. Ethics for this study was granted by the University of Otago Ethics Committee (reference number 13/085).

Eligible participants for this study were identified using a multifaceted approach. Restorative proctocolectomy with IPAA recipients were discovered from the Christchurch Public Hospital clinical coding department, the surgical records of Christchurch public hospital colorectal surgeons, the Christchurch private hospitals’ (Southern Cross Hospital and St. George's Hospital) patient databases, the Canterbury IBD clinical database established in 2006,^[21]^ gastroenterological and surgical colleague referrals, and self-referral through advertisements in public and private clinic waiting rooms. Complete capture of the population base was ensured by this multifaceted approach.

### 2.2 Outcomes

#### 2.2.1 Participant indications and complications

Participants’ medical records were accessed from primary care, specialist outpatient clinics, and all inpatient episodes from the point of restorative proctocolectomy with IPAA to the end of the study period. Data collected included demographics, the indication for restorative proctocolectomy with IPAA, and early and late complications. Underlying conditions included Crohn's disease, ulcerative colitis, indeterminate colitis, FAP, and Lynch syndrome. The indications were recorded as failed medical therapy, acute complications, dysplasia, FAP, or other.

Complications were divided into early (≤30 days after restorative proctocolectomy with IPAA) or late (>30 days after restorative proctocolectomy with IPAA). Early complications included hemorrhage requiring transfusion, wound infection, pelvic sepsis, and small bowel obstruction. Pelvic sepsis was defined as an “infective process in the peripouch area, detected during the investigation of clinical symptoms” and comprises all abscesses with or without anastomotic leak.^[6]^ Early complications were classified according to the Clavien–Dindo classification.^[15,16]^ Late complications included small bowel obstruction, pouchitis (diagnosed clinically and/or histologically), abscess or fistula, perianal stricture, and pouch failure. Perianal stricture was defined as a clinically significant stricture that required dilating in theatre; routine dilation of the anastomosis within 8 weeks postsurgery was not classified as stricture.

#### 2.2.2 Quality of life

QoL was measured via the IBDQ. The IBDQ^[11]^ contains 32 items divided into 4 health subdimensions: bowel symptoms, systemic symptoms, social function, and emotional function. Responses were scored on a 7-point Likert scale where 7 corresponds to the best function and one to the worst. Those who did not complete at least 31 questions on the IBDQ were considered as noncompleters.

### 2.3 Statistical methods

IBM SPSS Statistics for Windows, Version 22.0 (Armonk, NY: IBM Corp) was used for statistical analyses. Frequencies, percentages, means, and standard deviations were calculated for demographics and indications. Completers were compared to noncompleters. Early and late complication frequencies were determined. Odds ratios (ORs) with 95% confidence intervals (CIs) were then used to test for predictors of early and late complications generally as well as pouchitis and pouch failure specifically. Results were considered statistically significant if *P* < 0.05.

IBDQ at follow-up was calculated for all completers and comparisons were made for a number of variables. *t* Tests for independent means and 1-way analyses of variance were performed for demographics and complications. For missing data in the IBDQ, imputation was performed if one answer was missing; otherwise, the IBDQ was considered incomplete.

## 3 Results

### 3.1 Participant identification, eligibility, and consent

**Figure 1:**
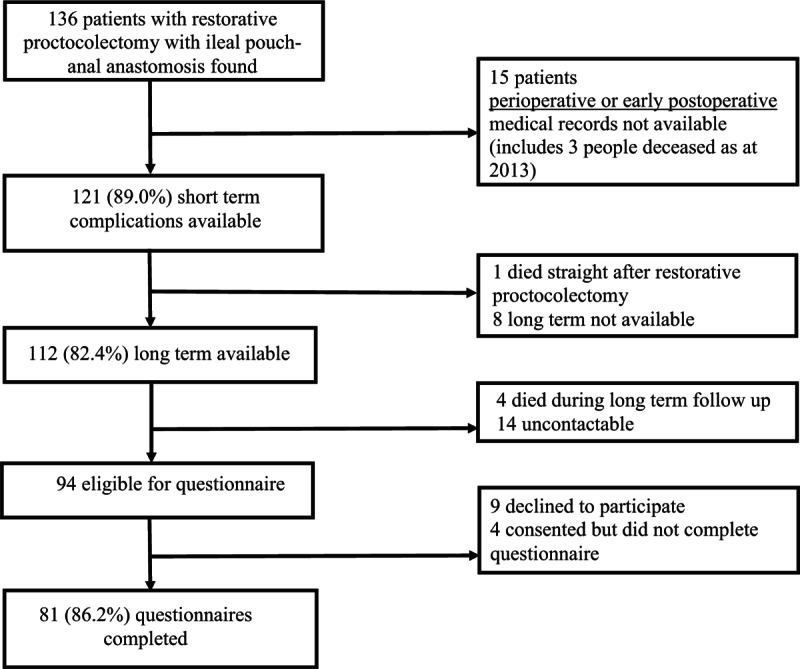
Participant identification and recruitment.

The final number of eligible participants for this study was 136 of whom 121 (89%) had early complication data available via perioperative or early postoperative medical records (Fig. 1). One patient died 3 weeks after restorative proctocolectomy with IPAA and 8 patients were lost leaving 112 (82%) available for long-term follow-up. Overall, 94 people were eligible for the questionnaire through being alive and contactable and 81 completed the IBDQ (86% response rate).

### 3.2 Demographics and indications

The demographics and indications of the 81 completers are shown in Table 1. The 81 completers were older on average than the 13 noncompleters (51.4 vs 42.8, *P* = 0.03). No such differences were found for age at restorative proctocolectomy with IPAA, years since surgery, diagnosis (Crohn's disease vs non-Crohn's disease), ethnicity, indication (acute vs elective), or presence of a stoma.

**Table 1:**
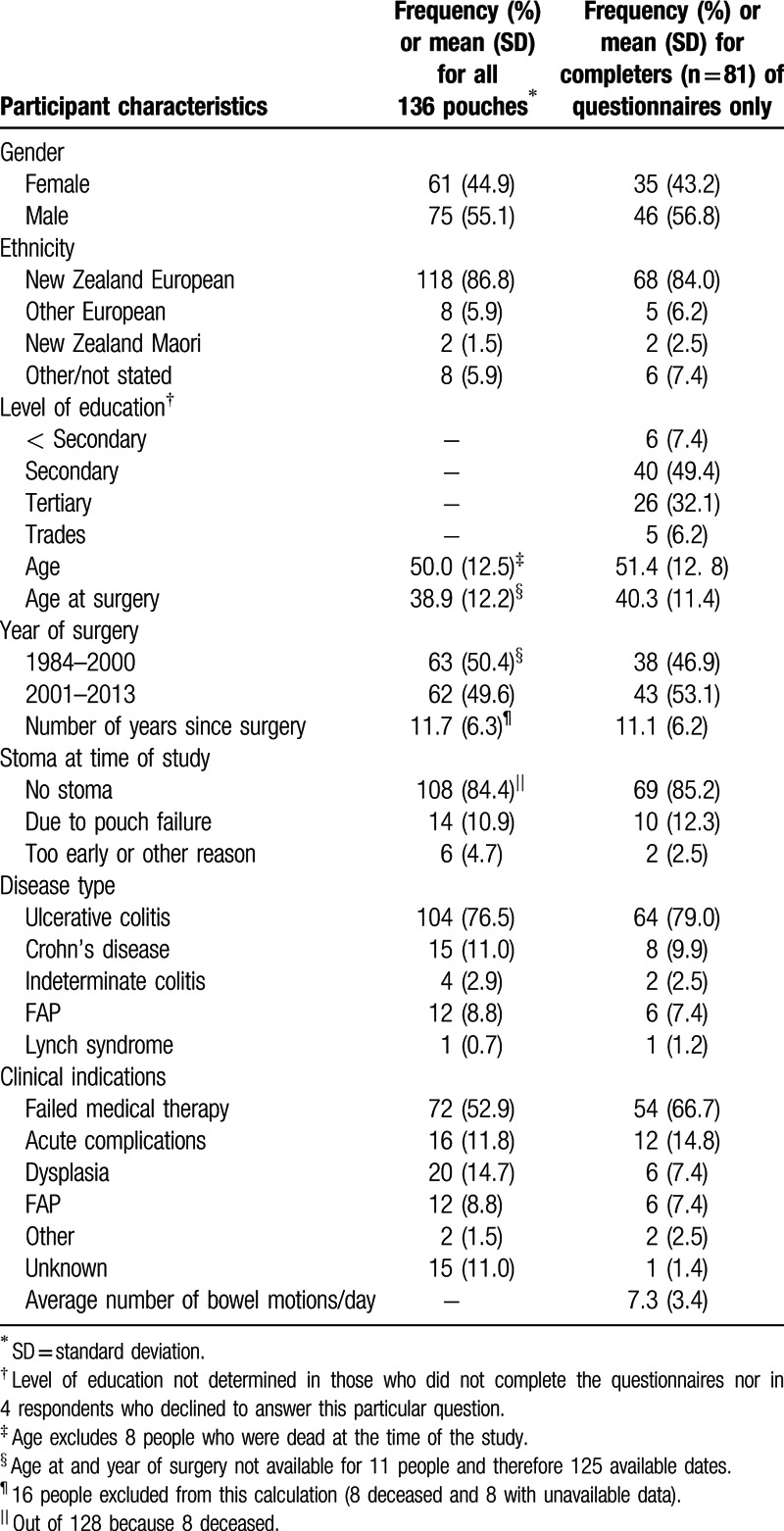
Participant demographics and indications.

### 3.3 Complications

Table 2 shows the early and late complication rates of the eligible participants with available data and IBDQ completers. Twenty-five point 6% had at least 1 early complication, whereas 76% had at least 1 late complication. The late complication rate dropped to 54% when pouchitis was excluded. Pouchitis was the most common complication, occurring in more than half of the participants at long-term follow-up. One patient died 3 weeks after surgery from liver failure on a background of diabetes and near end stage hepatic failure resulting from primary sclerosing cholangitis.

**Table 2:**
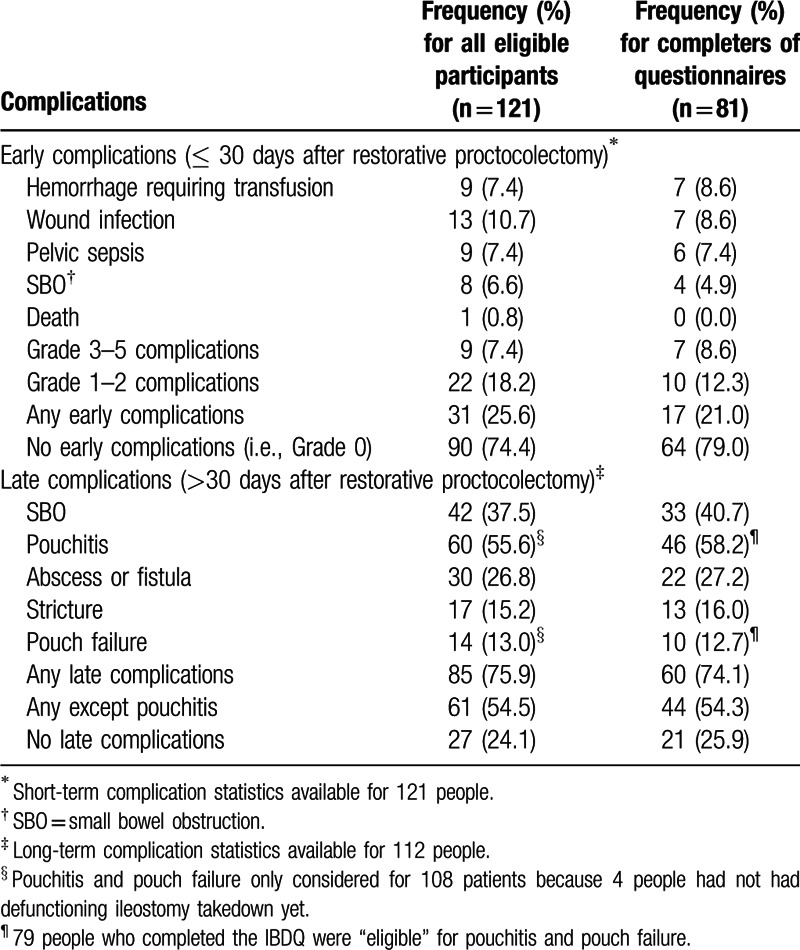
Complications.

### 3.4 Predictors of complications

Table 3 shows the ORs for predictors of early and late complications as well as pouch failure. Those aged more than 50 were less likely to have any early complication (OR = 0.32; 95% CI = 0.13–0.79). Those patients 12 or more years postsurgery were also more likely to have had a late complication (OR = 2.69; 95% CI = 1.07–6.77). Abscess or fistula (OR = 9.25; 95% CI = 2.62–32.62), stricture (OR = 6.30; 95% CI = 1.81–21.88), and a final diagnosis of Crohn's disease (OR = 5.87; 95% CI = 1.41–24.37) were all significantly associated with pouch failure.

**Table 3:**
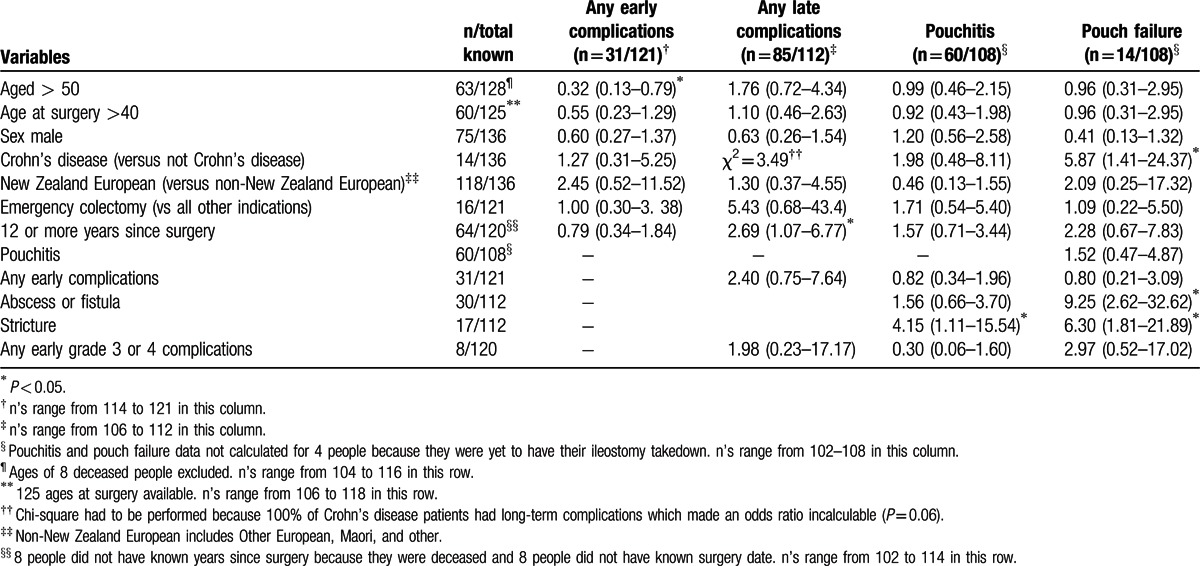
Odds ratios (and 95% confidence intervals) of predictors of any early or late complications, or pouchitis.

### 3.5 QoL at follow-up

The mean IBDQ score was 170.3 (standard deviation = 28.3). Table 4 shows the average IBDQ scores for all relevant independent variables, including early and late complications. Experiencing grade 3 or 4 early postoperative complications was associated with a 37-point decrease in IBDQ scores and this was significant (*P* = 0.001).

**Table 4:**
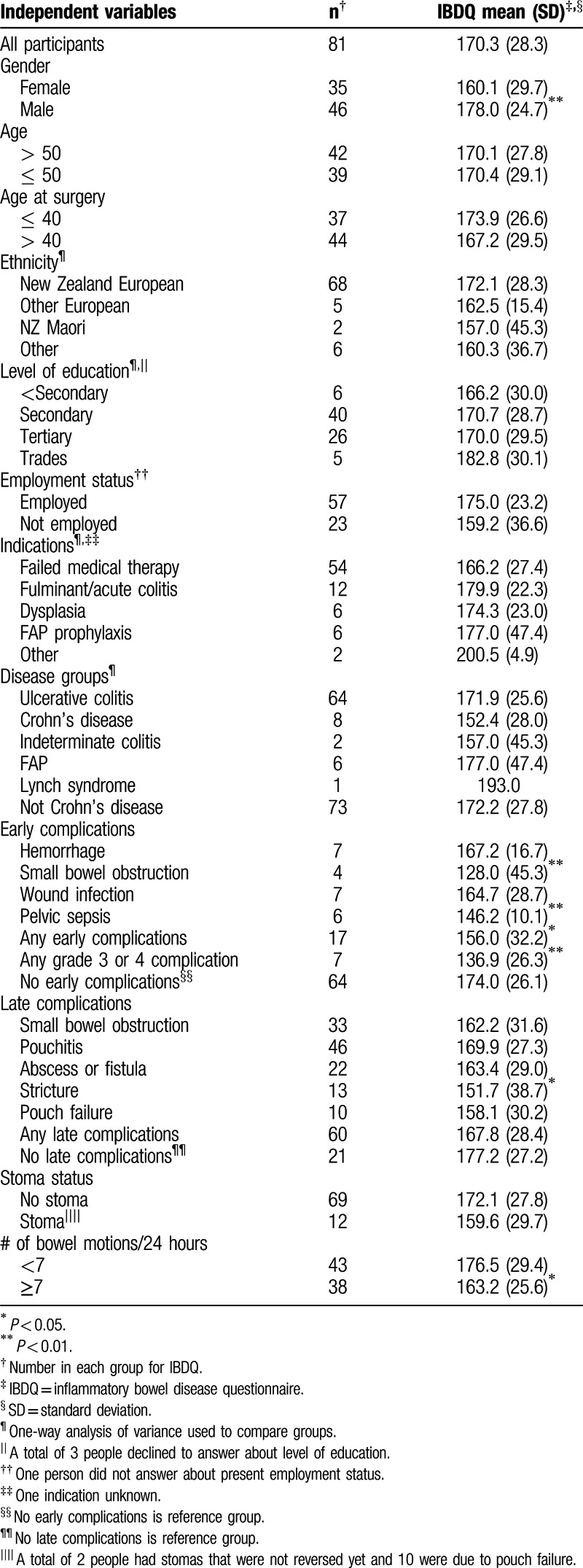
Quality of life and all independent variables at follow-up.

## 4 Discussion

This study aimed to determine if serious (Clavien–Dindo grades 3 and 4) early postoperative complications predicted pouch failure and impaired QoL in the long term. Those who had Clavien–Dindo grade 3 or 4 early postoperative complications had the worst QoL. While this classification has not previously been related to long term QoL in restorative proctocolectomy with IPAA recipients, 2 previous studies reported serious early postoperative complications (i.e., pelvic sepsis) to be associated with poorer QoL in the long term.^[15,16]^ This study reinforces the long term deleterious effects of serious early complications on functional outcomes of restorative proctocolectomy with IPAA and provides further motivation to minimize their occurrence. Factors associated with early postoperative complications need to be addressed at a surgeon and institutional level (such as volume) in order to maximize patients long-term QoL.

The restorative proctocolectomy with IPAA performed in Canterbury had early complication rates comparable to previous studies in Cleveland^[6]^ and the United Kingdom.^[4]^ Late complications are likely underreported and unreliable in many studies reported by quaternary institutions due to inability to follow-up patients who return to regional follow-up facilities. Fazio et al^[6]^ reported a 29% late complication rate, but excluded pouchitis and had a 90 day as opposed to a 30 day cut-off for early versus late complications. In the present study, the late complication rate fell to 55% when pouchitis was excluded. However, the present study had a longer median follow-up (12 years), was performed in a region where there is one major public hospital, and incorporated records from primary care. Therefore, the long-term complication rates in this study should be more accurate than those reported from quaternary centers.

The pouch failure rate was 13%. This is similar to other studies which have reported failure rates as low as 5% at median follow up of 84 months^[6]^ and as high as 16% at 10 years.^[4]^ As expected, Crohn's disease patients were far more likely to have a failed restorative proctocolectomy with IPAA than non-Crohn's disease patients adding further weight against the use of restorative proctocolectomy with IPAA in Crohn's disease patients.^[6–8]^ A previous study has suggested septic complications, defined as leaks, abscesses, or fistulas, are associated with restorative proctocolectomy with IPAA failure.^[22]^ The present study found abscess or fistulas to be strongly associated with pouch failure, as were strictures.

While it may be anticipated that pouch failure would be associated with reduced QoL, those with pouch failure in this study who had reverted to an ileostomy did not have significantly inferior QoL. Historically it has been suggested that restorative proctocolectomy with IPAA provides a better QoL than a permanent ileostomy. However, a recent systematic review challenged this assumption, finding restorative proctocolectomy with IPAA and end ileostomy have equivalent QoL.^[23]^ The present study extends that finding suggesting QoL is satisfactory with a permanent ileostomy even after restorative proctocolectomy with IPAA failure.

Despite the significant complication rates associated with restorative proctocolectomy with IPAA, overall QoL was satisfactory in this study. The mean IBDQ score was 170.3 which is what clinically stable IBD patients score on average.^[13]^ This is consistent with previous work reporting that QoL is comparable to the general population after restorative proctocolectomy with IPAA.^[24,25]^

The two main limitations of this study were the retrospective nature of analyzing indications and complications, with some notes not being available, and that 14 people were uncontactable. Nevertheless, the response rate among contactable and eligible people was high (86%).

## 5 Conclusion

Patients who experienced serious early postoperative complications had long-term impaired QoL. Nevertheless, the overall QoL for restorative proctocolectomy with IPAA recipients is good and patients with failed restorative proctocolectomy with IPAAs did not have significantly lower QoL than those with intact restorative proctocolectomy with IPAAs.
